# Roles of small RNAs in crop disease resistance

**DOI:** 10.1007/s44154-021-00005-2

**Published:** 2021-08-18

**Authors:** Jun Tang, Xueting Gu, Junzhong Liu, Zuhua He

**Affiliations:** 1 National Key Laboratory of Plant Molecular Genetics, CAS Center for Excellence in Molecular Plant Sciences, Institute of Plant Physiology & Ecology, Chinese Academy of Sciences, Shanghai, 200032 China; 2 University of the Chinese Academy of Sciences, Beijing, 100049 China; 3 State Key Laboratory of Conservation and Utilization of Bio-Resources in Yunnan and Center for Life Sciences, School of Life Sciences, Yunnan University, Kunming, China

**Keywords:** Small RNAs, HIGS, Crop diseases, RNAi, RNAi-based technology

## Abstract

Small RNAs (sRNAs) are a class of short, non-coding regulatory RNAs that have emerged as critical components of defense regulatory networks across plant kingdoms. Many sRNA-based technologies, such as host-induced gene silencing (HIGS), spray-induced gene silencing (SIGS), virus-induced gene silencing (VIGS), artificial microRNA (amiRNA) and synthetic *trans*-acting siRNA (syn-tasiRNA)-mediated RNA interference (RNAi), have been developed as disease control strategies in both monocot and dicot plants, particularly in crops. This review aims to highlight our current understanding of the roles of sRNAs including miRNAs, heterochromatic siRNAs (hc-siRNAs), phased, secondary siRNAs (phasiRNAs) and natural antisense siRNAs (nat-siRNAs) in disease resistance, and sRNAs-mediated trade-offs between defense and growth in crops. In particular, we focus on the diverse functions of sRNAs in defense responses to bacterial and fungal pathogens, oomycete and virus in crops. Further, we highlight the application of sRNA-based technologies in protecting crops from pathogens. Further research perspectives are proposed to develop new sRNAs-based efficient strategies to breed non-genetically modified (GMO), disease-tolerant crops for sustainable agriculture.

## Introduction

In nature, plants constantly face diverse biotic stresses, including bacteria, fungi, oomycetes, nematodes and viruses. Pathogen infection causes approximately 30% of global crop losses annually worldwide. Therefore, disease control is vital for assuring food security worldwide. During long coevolution with pathogens, plants have armed with various defense tools to prevent pathogens, which constitute a two-tiered immune machinery to detect and prevent pathogen invasion. The first layer is pathogen-associated molecular pattern (PAMP)-triggered immunity (PTI) governed by cell surface pattern recognition receptors (PRRs). In order to circumvent PTI, pathogens evolve effector proteins to suppress host PTI response, which is known as effector triggered susceptibility (ETS). As a counter-defense to prevent further infection, plants have then evolved highly polymorphic nucleotide-binding site (NBS) and leucine-rich repeat (LRR) domain-containing (NLR) resistance (R) proteins that directly or indirectly recognize pathogen effectors and form homo- or hetero-NLR complexes or resistosomes to trigger effector-triggered immunity (ETI) (Alves et al. 2014; Cui et al. 2015; Dangl et al. 2013; Ma et al. 2020; Spoel and Dong 2012; Wang et al. 2019a, b; Zhou and Zhang 2020). The second layer of immunity is more robust than PTI, which usually confers high resistance against pathogens. Therefore, NLR immune receptors are the major breeding targets for disease resistance in crops (Li et al. 2020a; Deng et al. 2020). PTI and ETI are interconnected and play synergistic roles to induce a set of downstream defense responses such as the generation of reactive oxygen species (ROS) and global transcriptional reprogramming for defense (Ngou et al. 2021; Yuan et al. 2021).

Small RNAs (sRNAs) are a class of 18–30 nt, non-coding RNAs, which play vital roles in regulating gene expression and maintaining the genome stability. Based on the different biogenesis pathway and the divergent modes of action, plant sRNAs can be cataloged into two major classes, microRNAs (miRNAs) and small interfering RNAs (siRNAs). MiRNAs and siRNAs are generated from miRNA genes (*MIRs*) and double-stranded RNAs (dsRNAs) by the cleavage activity of Dicer-like (DCL) proteins, respectively (Borges and Martienssen 2015; Chen 2009; Ghildiyal and Zamore 2009). Plant primary miRNAs (pri-miRNAs) are transcribed from *MIR*s by RNA polymerase II (Pol II) and trimmed into precursor miRNAs (pre-miRNAs) by DCL1, and pre-miRNAs are further processed by DCL1 to generate mature miRNAs (Kurihara and Watanabe 2004). Mature miRNAs, after stabilized by HEN1, a methyltransferase that catalyzes 2′-O-methylation, are loaded onto ARGONAUTE (AGO) proteins and other components to form the RNA-induced silencing complex (RISC) to silence targets by mRNAs cleavage or translational inhibition at post-transcriptional level, or directing DNA methylation that represses transcription at transcriptional level (Padmanabhan et al. 2009; Song et al. 2019; Voinnet 2009; Wu et al. 2010). siRNAs in plants are generated by DCL2-4 from diverse endogenous and exogenous dsRNAs precursors. An expanding world of plant siRNAs has been discovered, such as heterochromatic siRNAs (hc-siRNAs), phased, secondary siRNAs (phasiRNAs) and natural antisense siRNAs (nat-siRNAs) (Baulcombe 2004; Bologna and Voinnet 2014; Borges and Martienssen 2015; Castel and Martienssen 2013; Fei et al. 2013; Ghildiyal and Zamore 2009). Hc-siRNAs are derived from single-stranded RNA (ssRNA) transcribed from transposable elements and other repeat regions by Pol IV. The ssRNA transcripts are converted into dsRNAs by RNA-dependent RNA polymerase 2 (RDR2), and hc-siRNAs are subsequently produced by DCL3-mediated processing (Li et al. 2006; Pontes et al. 2006). The hc-siRNAs can direct DNA methylation in an AGO4-dependent pathway, which is known as RNA-directed DNA methylation (RdDM) (Chan et al. 2004). phasiRNA biogenesis requires the activity of miRNA-containing RISC, which cleaves the transcripts derived from phasiRNA-producing loci (*PHAS* loci). After cleavage, the transcripts are converted to dsRNAs, which produce 21- or 24-nt phasiRNAs by the activity of DCLs (Fei et al. 2013). Trans-acting siRNAs (tasiRNAs) are the first reported phasiRNAs in plants. A noncoding transcript originated from tasiRNA locus (namely *TAS* genes) that is converted into dsRNAs by RDR6 and then dsRNAs are cleaved into 21-nt siRNAs by DCL4 (Gasciolli et al. 2005; Peragine et al. 2004). phasiRNAs can be loaded onto AGOs and exert their repressive roles to their targets in *trans* (Fei et al. 2013). Nat-siRNAs are produced by the activity of DCL1/2, RDR6 and HEN1 from dsRNA precursors, which originate from bidirectional transcription of two partially overlapping genes (cis-natsiRNAs) or highly complementary transcripts derived from different loci in the genome (trans-natsiRNAs) (Katiyar-Agarwal et al. 2006). These siRNAs, like miRNAs, also play essential regulatory roles in plant development and abiotic/biotic stress by modulating target gene expression either transcriptionally through RdDM or post-transcriptionally by cleaving target mRNAs and/or repressing gene translation (Bologna and Voinnet 2014; Borges and Martienssen 2015; Castel and Martienssen 2013; Ghildiyal and Zamore 2009). Therefore, sRNAs actively regulate gene and protein expression, thereby imposing important impacts on various physiological processes.

In the following section, we will summarize recent findings and current progresses on sRNA involvement and roles in crop disease resistance, with aspects of sRNAs in crop-fungi, crop-oomycetes, crop-bacterial, and crop-virus interactions. We then propose research perspectives in sRNAs-based technologies in crop disease control.

## Overview of sRNAs involved in PTI and ETI

**Fig. 1:**
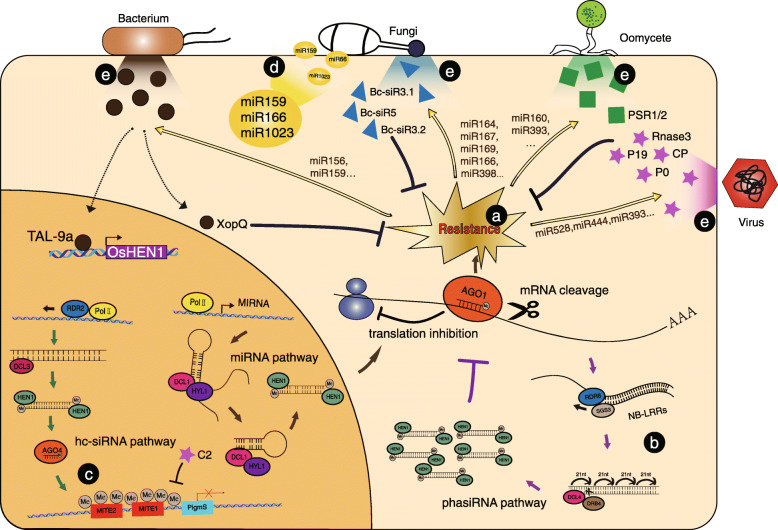
Small RNAs regulate plant defense against-pathogen. In response to pathogen attacks, plants accurately fine-tune the expression of endogenous gene through sRNAs-trigged mRNA cleavage, translational repression as well as DNA methylation. **a** sRNAs regulate plant resistance to various pathogens through mRNA cleavage or translational repression. miR164 (Wang et al. 2018), miR167 (Zhao et al. 2020) and miR169 (Li et al. 2017) which directly target transcription factors play negative roles in plant response to *M. oryzae* while miR166 (Salvador-Guirao et al. 2018) and miR398 (Li et al. 2019a) are positive regulators. miR160 (Natarajan et al. 2018) and miR393 (Wong et al. 2014) regulate the interactions between crop and oomycetes. Some other small RNAs such as miR156 (Liu et al. 2019) and miR159 (Zhao et al. 2015b) modulate the disease resistance against bacterial pathogens. Upon virus infection, some miRNAs target essential components of RNAi to regulate plant immunity, like miR168 (Du et al. 2011). **b** Hc-siRNA-mediated DNA methylation at the MITE region of *PigmS* promoter represses *PigmS* expression (Deng et al. 2017). **c** Plant small RNAs not only function in host cells, but also move into invasive enemies. For example, miR159, miR166 (Zhang et al. 2016c) and miR1023 (Jiao and Peng 2018) trigger the silencing of fungal virulence genes. **d** PhasiRNA pathway fine-tunes the expression level of R gene in the absence of pathogen (González et al. 2015). **e** During the long-term arm-race between crops and pathogens, pathogens can secrete specific proteins or small RNAs into plant cells to enhance plant susceptibility to ensure their own virulence. For instance, *B. cinerea* delivers Bc-siRNAs to plant cell to hijack host RNAi pathway (Weiberg et al. 2013). TAL-effectors such as Tal9a from genus *Xanthomonas* can bind to host specific promoter motifs and activate host genes expression (Moscou and Bogdanove 2009). A non-TAL effector XopQ can up-regulates host sRNAmiR1876 through an unknown mechanism (Jiang et al. 2020). Some proteins such as PSR1 and PSR2 (Xiong et al. 2014) from *P. sojae* and P19 (Silhavy et al. 2002), P0 (Li et al. 2019b) , CP (Karran and Sanfaçon 2014), RNase 3 (Cuellar et al. 2009; Kreuze et al. 2005), C2 (Yang et al. 2013) from different kinds of viruses can disrupt host immune response by suppressing RNA silencing pathways in plants

Accumulative evidences have uncovered important roles of miRNAs in regulating immune responses in plants (Fei et al. 2016; Huang et al. 2019; Katiyar-Agarwal and Jin 2010; Padmanabhan et al. 2009; Ruiz-Ferrer and Voinnet 2009; Wang & Galili 2019; Wang et al. 2019a, b; Weiberg et al. 2014) (Fig. 1a). The first miRNA reported to modulate plant immunity is Arabidopsis miR393. miR393 is induced by the PAMP peptide flg22 and guides the cleavage of the mRNAs coding the F-box auxin receptors TIR1, AFB2, and AFB3 to attenuate the auxin signaling pathway, which is required for PTI response (Navarro et al. 2006). In addition to miR393, miR167 and miR160, induced by *Pseudomonas syringae* pv. *tomato* DC3000 *hrcC* that triggers PTI but not ETI in Arabidopsis, also target auxin-response factors (ARFs) to regulate auxin signaling (Zhang et al. 2011). Upon flg22 treatment, miR160a, miR398b and miR773 regulated callose deposition (Li et al. 2010).

More importantly, miRNAs fine-tune the transcription of NLR immune receptors to efficiently balance defense and growth in plants (Fei et al. 2016; Huang et al. 2019; Wang et al. 2019a, b), since at the absence of pathogens, high expression of *NLR* genes may result in the fitness cost in plants (Purrington 2000; Richard et al. 2018; Tian et al. 2019). miRNAs such as miR482/2118 family (Fei et al. 2016), Bra-miR1885 (He et al. 2008), id47, id97, id113 (Carra et al. 2009), Pta-miR946 (Lu et al. 2007), and Md-miRLn11 (Ma et al. 2014) mediate cleavage of NLR transcripts in Arabidopsis, *Brassica rapa*, *Vitis vinifera*, *Pinus taeda*, *Citrus trifoliata* and woody plants, respectively.

Besides miRNAs, siRNAs also function as key regulators of plant immunity (Fig. 1a). For example, nat-siRNAATGB2, which originate from the overlapping region of transcripts of a small GTP-binding protein *ATGB2* and a pentatricopeptide repeat-like (*PPRL*) gene, is induced upon infection by *P. syringae* carrying effector *avrRpt2*, and silences *PPRL* that negatively regulates *RPS2*-mediated disease resistance (Katiyar-Agarwal et al. 2006). miR482/miR2118-mediated phasiRNAs can also fine-tune the expression of the cognate NLR genes in divergent plant species such as Arabidopsis (Borrelli et al. 2018), tomato (*Solanum lycopersicum*) (Canto-Pastor et al. 2019), Medicago (*Medicago truncatula*) (Zhai et al. 2011) and soybean (*Glycine max*) (Zhao et al. 2015a). In the absence of pathogens, the miR482/2118-phasiRNA cascade favors growth by suppressing NLR expression (Fig. 1b). Upon pathogen infection, the phasiRNAs-mediated suppression of NLRs is released to ensure ETI (González et al. 2015). In *dcl4* and *ago1* mutant plants, the NLR gene *SNC1* is constitutively activated and multiple R genes in *RPP5* locus are upregulated (Yi and Richards 2007).

## sRNAs modulate resistance against fungal pathogens in crops

Fungal pathogens are a major group of plant invaders that cause many notorious plant diseases. Among them, *Magnaporthe oryzae* (*M. oryzae*) causes rice (*Oryza sativa*) blast and is the most destructive fungal pathogen worldwide (Dean et al. 2012; Zhang et al. 2016b). Several miRNAs have been reported to negatively affect blast resistance. These miRNAs usually mediate transcriptional reprogramming to regulate immune responses through directly targeting transcription factors (Fig. 1a). For example, Osa-miR164a targets OsNAC60 and negatively regulates its activity, which suppresses rice blast resistance. Moreover, the Osa-miR164a/NAC60 module plays a conserved regulatory role in plant resistance to rice sheath blight, tomato late blight, and soybean root and stem rot diseases (Wang et al. 2018). Osa-miR169 suppresses transcription factor NF-YAs and enhances rice susceptibility to *M. oryzae* (Li et al. 2017). Similarly, Osa-miR167d also negatively regulates rice immunity against *M. oryzae* (Zhao et al. 2020). When infected by *M. oryzae*, rice plants accumulate higher levels of Osa-miR319, which guides cleavage of *OsTCP21* mRNA*.* The Osa-miR319-mediated suppression of *OsTCP21* results in stronger disease symptoms by reducing cellular ROS and jasmonic acid (JA) levels (Zhang et al. 2018). miR396 is a highly conserved miRNA family targeting *Growth Regulating Factor* (*OsGRF*) genes. The miR396-*OsGRF* module plays a vital role in balancing growth and immunity against the blast fungus. Overexpressing of a miR396-resistant version of *OsGRF* or blocking miR396 expression not only enhances rice resistance to *M. oryzae* but also improves yield traits (Chandran et al. 2018). A target mimic of miR156fhl-3p (MIM156-3p) indirectly increases the expression of squamosa promoter-binding-like transcription factor *OsSPL14* to enhance rice blast disease resistance by reducing the abundance of miR156-5p, which fine-tunes the tradeoff between blast disease resistance and yield (Zhang et al. 2020). Most recently, Osa-miR439 is reported to negatively regulate rice blast resistance through inhibiting the induction of defense-related genes and accumulation of H_2_O_2_ (Lu et al. 2021).Osa-miR168 can target OsAGO1 and suppression of Osa-miR168 by a target mimic (*MIM168*) improves yield, flowering time and immunity to *M. oryzae,* which suggest the potential application of miRNAs in coordinating plant immunity with growth and development (Wang et al. 2021). Interestingly, some miRNAs play positive roles in rice blast resistance. Overexpression of miR160a in transgenic rice plants enhances resistance to the blast fungus. Overexpressing *MIR166k-h* modulates *EIN2* expression and therefore enhances rice resistance to *M. oryzae* and *Fusarium fujikuroi* (Salvador-Guirao et al. 2018). Osa-miR398b suppresses the expression of several targets, *Cu/Zn-Superoxidase Dismutase* (*CSD1/2*), *Superoxide Dismutase X* (*SODX*), and *Copper Chaperone for Superoxide Dismutase* (*CCSD*). *csd1/2* and *sodx* mutants display increased H_2_O_2_ accumulation and enhanced blast resistance, while *ccsd* mutants show enhanced blast susceptibility with lower levels of H_2_O_2_ (Li et al. 2019a). A novel rice miRNA, Osa-miR7695, positively regulates resistance to *M. oryzae* by targeting *Natural resistance-associated macrophage protein 6* (*OsNramp6*), a Fe transporter. Interestingly, Osa-miR7695 appears to be subjected to subspecies-specific selection. Overexpression of Osa-miR162a induces defense genes expression and the accumulation of H_2_O_2_, and increases blast resistance in transgenic rice (Li et al. 2020b).

Importantly, the RdDM pathway plays an important role in regulating immune responses against the blast fungus (Deng et al. 2017) (Fig. 1c). In rice, *Pigm* locus fine-tunes blast resistance and the trade-off between defense and yield. Among NLRs of the *Pigm* cluster, PigmR confers broad-spectrum resistance, whereas PigmS, whose expression is modulated by RdDM pathway, competitively suppresses PigmR homodimerization to suppress resistance. RNAi-mediated silencing of the RdDM pathway genes, *OsRDR2*, *OsDCL3a*, and *OsAGO4a*, in Pigm background reduces hc-siRNA accumulation and methylation levels of CHH sites at the MITE (Miniature Inverted-Repeat Transposable Elements) region of *PigmS* promoter and subsequently facilitates *PigmS* expression level (Deng et al. 2017). Blocking the expression of OsAGO4a in *Pigm* background increases blast susceptibility, consistent with the attenuation of *PigmR*-mediated resistance by *PigmS* (Deng et al. 2017). This DNA methylation-mediated regulation of the PigmR-PigmS NLR pair might provide a molecular niche that alleviates selection pressure for mutations against PigmR recognition, ensuring durable resistance to *M. oryzae*. Mostly recently, a novel MITE-derived microRNA, Osa-miR812w, is reported to positively regulate rice blast resistance through directing DNA methylation at target genes in *cis* and *trans* (Campo et al. 2021). In addition to *M. oryzae*, some sRNAs regulate the defense against other fungal pathogens in rice. The necrotrophic fungal pathogen *Rhizoctonia solani* (*R. solani*) represses the expression of siR109944 in rice. One candidate target of siR109944 is F-Box domain and LRR-containing protein 55 (FBL55), which is a transport inhibitor response 1 (TIR1)-like protein. Transgenic plants disrupting siR109944 biogenesis or overexpressing *FBL55* display enhanced resistance to *R. solani*, probably attributing to the altered auxin homeostasis by FBL55 (Qiao et al. 2020). In wheat (*Triticum aestivum* L.), miR408 positively regulates plant resistance to wheat stripe rust fungus by guiding the cleavage of *TaCLP1* mRNA (Feng et al. 2013). In barley (*Hordeum vulgare* L.), a novel miR9863 family triggers the biogenesis of 21-nt phasiRNAs and miR9863-phasiRNA cascade forms a feed-forward regulatory machinery to suppress the immune signaling mediated by group I *Mildew resistance locus a* (*Mla*) alleles in response to barley powdery mildew fungus. Overexpression of miR9863 members specifically attenuates disease resistance and cell death triggered by MLA1 but not MLA10 (Liu et al. 2014). Interestingly, *Mla* as well as *Rom1* (*restoration* of *Mla resistance 1*) negatively regulate accumulation levels of hvu-miR398 which represses *HvSOD1* accumulation and influences ETI in response to the powdery mildew fungus (Xu et al. 2014). In cotton (*Gossypium hirsutum* L.), the ghr-miR477-silencing lines display decreased resistance to *Verticillium dahlia*, while knockdown of its target CaM-binding protein *GhCBP60A* increases plant resistance by up-regulating isochorismate synthase *GhICS1* expression to increase salicylic acid (SA) level (Hu et al. 2020).

Surprisingly, recent studies have revealed that sRNAs can move within plant cells through plasmodesmata and phloem, and transport between plant cells and pathogens by vesicles (Cai et al. 2019; Cai et al. 2018a; Chitwood and Timmermans 2010; Devers et al. 2020; Dunoyer et al. 2010). The bidirectional cross-kingdom movements of sRNAs between plants and pathogens have two different effects on plant immunity. On one hand, some host sRNAs can be transported into invading pathogens and suppress their virulence (Fig. 1d). Upon the infection of *Verticillium dahliae*, the production of cotton miR166 and miR159 is increased, and both miRNAs are transported to the fungus to specifically silence virulence genes, *Ca*^*2+*^*-dependent cysteine protease* (*Clp-1*) and *isotrichodermin C-15 hydroxylase* (*HiC-15*), respectively (Zhang et al. 2016c). Similarly, wheat miR1023 can silence the alpha/beta hydrolase gene of *F. graminearum* to suppress its invasion (Jiao and Peng 2018). These miRNAs may be secreted by exosome-like extracellular vesicles at the infection sites and taken up by the fungal cells to induce silencing of fungal genes associated with pathogenicity, similar to the role of sRNAs (TAS1c-siR483, TAS2-siR453, and IGN-siR1) secreted by Arabidopsis cells in defense against *Botrytis cinerea* (*B. cinerea*) (Cai et al. 2018a, b). Above all, the export of specific host miRNAs and siRNAs to inhibit the expression of virulence genes in pathogens may be a conserved and efficient host defense strategy against fungal pathogens.

On the other hand, some sRNAs can serve as effectors to suppress host immunity by hijacking host RNA interference pathways (Fig. 1e). After *B. cinerea* infection, *mitogen activated protein kinase 1/2* (*MPK1/2*), *peroxiredoxin* (*PRXIIF*) and *cell wall-associated kinase* (*WAK*) mRNAs, are targeted by Bc-siR3.2, Bc-siR3.1 and Bc-siR5, respectively. *B. cinerea dcl1 dcl2* double mutant that cannot produce Bc-sRNAs displays reduced pathogenicity (Weiberg et al. 2013), whereas *dcl1* or *dcl2* single mutant still produces sRNA effectors to maintain virulence on plants, supporting that sRNA effectors are essential for *B. cinerea* pathogenicity (Wang et al. 2016b; Weiberg et al. 2013). *B. cinerea* can also deliver small RNA effectors Bc-siR37 into host cells to suppress host immunity by targeting *AtWRKY7*, *AtPMR6*, and *AtFEI2* (Wang et al. 2017)*.* Fol-milR1 from *Fusarium oxysporum* f.sp. *lycopersici* (*Fol*), which is exported into tomato cells after infection and sequentially loaded onto tomato ARGONAUTE 4a (SlyAGO4a), targets the CBL-interacting protein kinase *SlyFRG4*. *slyfrg4* mutant plants exhibit enhanced disease susceptibility to *Fol*, while *slyago4a* knock-down plants display enhanced resistance to *Fol* (Ji et al. 2021)*.*

## sRNAs regulate the interaction between crops and oomycetes

Oomycetes, one of the two most important groups of eukaryotic plant pathogens, are classified in the kingdom *Protoctista* and are evolutionally related to heterokont, biflagellate, golden-brown algae (Thines 2018). Several recent reports have revealed the important regulatory roles of sRNAs in plant resistance to two important oomycetes, *Phytophthora infestans* (*P. infestans*) and *Phytophthora sojae* (*P. sojae*) (Fig. 1a).

In potato (*Solanum chacoense* and *Solanum tuberosum* cv. Désirée) plants infected by *P. infestans*, miR160 is induced in both local and systemic leaves. miR160 knockdown plants fail to elicit systemic acquired resistance (SAR). miR160 targets and mediates the cleavage of *StARF10* mRNA. *St*ARF10 protein can bind to the promoter of *StGH3.6*, a key hub in SA-auxin cross-talk, suggesting the important roles of miR160-StARF10-StGH3.6 module in the antagonistic cross-talk between SA-mediated pathogen defense processes and auxin-mediated growth (Natarajan et al. 2018). In soybean infected by *P. sojae*, several miRNAs, such as miR1510, miR393, miR1507 and miR2109, regulate plant defense responses. Upon the infection, miR1510 expression is repressed, along with the increased accumulation level of its target *Glyma.16G135500*, which encodes a classic type of plant disease resistance-associated gene. The overexpression of *gma-miR1510a/b* in hairy roots enhances susceptibility, suggesting that miR1510 may serve as a negative regulator in plant defense against *P. sojae*. However, miR393 confers enhanced resistance to *P. sojae* by up-regulating the expression of 2-hydroxyisoflavanone dehydratase *GmHID1* and isoflavone synthase *GmIFS1* in isoflavonoid biosynthetic pathway (Wong et al. 2014). miR1507 and miR2109 trigger the production of phasiRNAs to regulate the expression of defense-associated R genes during *P. sojae* infection (Wong et al. 2014). These RNA silencing pathways can be suppressed by two members of conserved RxLR family effectors, PSR1 and PSR2, which are secreted by *P. sojae* (Qiao et al. 2013; Qiao et al. 2015; Xiong et al. 2014) (Fig. 1e). Above all, our knowledge on the roles of sRNAs in regulating the crosstalk between host and oomycetes remains largely elusive. Further systemic studies are needed to uncover the potential regulatory roles of small RNAs in defending crops against important oomycetes pathogens.

## sRNAs alter crop resistance against bacterial pathogens

Diverse classes of plant endogenous sRNAs, including miRNAs and hc-siRNAs, have been reported to modulate crop immune responses to bacterial pathogens (Fig. 1a). The bacterial blight caused by *Xanthomonas oryzae* pv. *Oryzae* (*Xoo*), the most devasting vascular diseases in rice, causes severe crop loss each year. Downregulation of Osa-miR156 or overexpression of its targets, Ideal Plant Architecture1 (IPA1) and transcription factor OsSPL7, lead to enhanced resistance against *Xoo* at the expense of rice yield (Liu et al. 2019). To ensure rice resistance to *Xoo* without yield penalty, specific promoter is selected and applied in plant genetic engineering. In *Xanthomonas,* TAL (transcription activator-like) effectors bind to specific motifs in host gene promoters and activate the expression of host genes (Moscou and Bogdanove 2009). Among these effectors, Tal9a can bind to the promoter of *OsHEN1* and activate its expression, which increases the accumulation levels of many small RNAs and enhances plant susceptibility (Liu et al. 2019) (Fig. 1e). Overexpression of *IPA1* driven by *OsHEN1* promoter can fine-tune fitness penalty and disease resistance against *Xoo* (Liu et al. 2019). Besides, the expression of Osa-miR159 is repressed by *Xoo* invasion, which activates the expression of Osa-miR159 targets *GAMYB* and *OsLRR-RLK2* to modulate plant responses to *Xoo* (Zhao et al. 2015b)*.* Interestingly, in maize (*Zea mays*) treated by *Bacillus velezensis* FZB42, four miRNAs, Zma-miR169a-5p, Zma-miR169c-5p, Zma-miR169i-5p and Zma-miR395b-5p, are repressed, which might fine-tune the activity of NF-Y transcription factors to activate the induced systemic resistance (ISR) to enhance plant defense response against pathogen infections (Xie et al. 2019).

In addition to the posttranscriptional activity of miRNAs, some miRNAs and hc-siRNAs can regulate plant resistance to bacterial pathogens through RdDM pathways. During *Xoo* infection, *OsNBS8R* expression is induced by PAMPs, e.g., flagellin and chitin, which activates downstream signaling in PTI to increase rice resistance. However, the non-TAL effector XopQ from *Xoo* up-regulates Osa-miR1876 and suppresses the transcription of *OsNBS8R* through DNA methylation, which partly underlies the ETS in rice-*Xoo* arm-race (Jiang et al. 2020). TE-siR815, a hc-siRNA generated from *WRKY45-1* locus, reduces rice resistance to *Xoo* through repressing the expression of ST1 that encodes an LRR-type protein by RdDM. Suppression of ST1 abolishes WRKY45-mediated resistance, thereby leading to disease susceptibility (Zhang et al. 2016a).

## Diverse sRNAs participate in crop-viruses interaction

In the battle between plants and viruses, virus-derived siRNAs (vsiRNA) as well as plant endogenous virus-activated miRNAs or siRNAs are loaded onto antiviral AGOs to inhibit the invasion of DNA or RNA viruses. These siRNAs may repress viral RNA through mRNA cleavage or translational inhibition, silence viral DNA through RdDM pathway, or affect host resistance (Carbonell and Carrington 2015; Prasad et al. 2019).

Some miRNAs in rice, such as Osa-miR528, Osa-miR444 and Osa-miR393, have been reported to positively or negatively regulate virus pathogenicity or host resistance (Fig. 1a). Osa-miR528 can be activated by the transcription factor OsSPL9 and negatively regulate the expression of its target *L-Ascorbate Oxidase* (*AO*) mRNA in rice. Loss-of-function of *spl9* causes decreased Osa-miR528 accumulation and a substantial increase of *AO*, resulting in enhanced plant resistance to *Rice stripe virus* (*RSV*) (Wu et al. 2017). Osa-miR444 has a pivotal function in the cross talk of nitrate signaling and the antiviral response. Without *RSV* infection, the expression of *OsRDR1*, an important component in RNA silencing pathway, is repressed by *OsMADS23*, *OsMADS27a* and *OsMADS57*. Upon virus infection, Osa-miR444 is induced to diminish the expression of *OsMADS* members and subsequently activates the OsRDR1-dependent RNA silencing pathway, which confers plant resistance against virus infection by silencing both viral RNAs and host genes (Wang et al. 2016a). In addition to the antibacterial role (Navarro et al. 2006), Osa-miR393 also regulates plant antiviral responses by repressing auxin signaling. Transgenic rice plants overexpressing Osa-miR393 display increased susceptibility to *Rice black streaked dwarf virus* (*RBSDV*) due to the repression of auxin signaling (Zhang et al. 2019). In *Brassica*, miR1885 fine-tunes plant growth and immunity through different mechanisms. During vegetative stage, miR1885 is maintained at low levels to ensure normal development and basal immunity. After infected by *Turnip mosaic virus* (*TuMV*), silencing of *BraCP24* mediated by miR1885 is enhanced to initiate precocious flowering, whereas the repression of R gene *BraTNL1* by miR1885-phasiRNAs cascade is antagonized by *TuMV*-induced *BraTNL1* expression (Cui et al. 2020). In tobacco, miR-6019 and miR-6020 cleave *N* gene to generate phased siRNAs, which compromise resistance to *Tobacco mosaic virus* (*TMV*) by repressing the *N* gene expression under normal circumstances. *TMV* infection diminishes the accumulation levels of miRNA-6019/6020 and phasiRNAs, thus releasing the repression of *N* gene and limiting the virus spread (Deng et al. 2018). These reports have revealed the complicated roles of miRNAs and phasiRNAs in plant resistance against virus.

vsiRNAs or plant siRNAs-mediated RNAi pathways are major antiviral defense machinery in plant model systems. Among the essential components of RNAi, RDR6 acts as a positive regulator in the resistance against viruses. Upon *Rice Dwarf Phytoreovirus* (*RDV*) infection, the expression of RDR6 is downregulated. Besides, the accumulation of *RDV* vsiRNAs is reduced in the *osrdr6* knockdown transgenic plants, which results in increased susceptibility in rice (Hong et al. 2015). Besides RDR6*,* multiple AGOs function in crop antiviral defense, such as AGO1/18 in rice, AGO1/2/4 in *Nicotiana benthamiana* (Carbonell and Carrington 2015). In rice, AGO1 and AGO18 synergistically modulate antiviral defense. AGO18 induced by virus competes with AGO1 for binding Osa-miR168 to release miR168-mediated suppression of AGO1 upon viral infection. Overexpression of miR168-resistant AGO1 rescues the deficiency of *ago18* in viral resistance (Du et al. 2011).

Like oomycetes, fungal and bacterial pathogens, viruses have evolved multiple virulence or effector proteins to disrupt host immune responses or RNA silencing pathways (Fig. 1e). *Polerovirus* P0 interacts with E3 ligase S-phase kinase regulated protein 1 (SKP1) to enhance the degradation of multiple AGOs by 26S proteasome system and autophagy pathways before RISC assembly (Li et al. 2019b). *Tomato ringspot virus* (*ToRSV*) suppressor coat protein (CP) binds to AGO1, suppresses its translational inhibitory activity and further enhances AGO1 degradation through autophagy (Karran and Sanfaçon 2014). RNase 3, a VSR encoded by *Sweet potato chlorotic stunt crinivirus* (*SPCSV*), cuts 21-24-nt vsiRNAs into 14 bp inactive products, thus effectively precluding the formation of antiviral RISC (Cuellar et al. 2009; Kreuze et al. 2005). C2 protein, encoded by DNA virus *Beet severe curly top virus*, is an effector that counteracts antiviral defense by interfering with gene silencing and metabolic defense responses. C2 mediates a decrease in DNA methylation levels of promoter regions from where reduced siRNAs derived, thereby upregulating the expression of the viral coding genes (Yang et al. 2013). The virus 19 kDa protein (P19) of *tombusviruses* inhibits post-transcriptional gene silencing by specifically binding to double-stranded siRNAs in tobacco (Silhavy et al. 2002). VsiRNA1 can suppress the expression of wheat *thioredoxin-like* (*TaAAED1*) gene which negatively regulates the production of ROS (Liu et al. 2021). Transgenic amiRNA1 plants in wheat confers a broad-spectrum disease resistance to *Chinese wheat mosaic virus, Barley stripe mosaic virus*, and *Puccinia striiformis* f. sp. *tritici* (Liu et al. 2021).

## sRNA-based technologies in engineering disease resistance

**Fig. 2:**
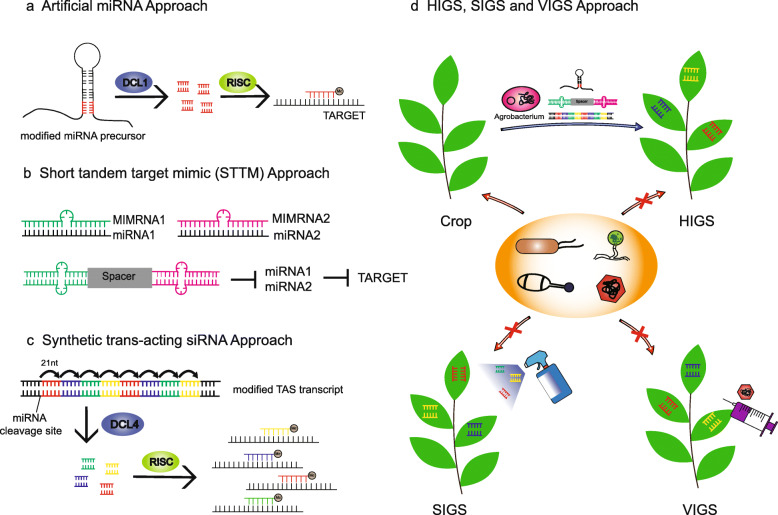
sRNA-based technologies in protecting crop plants. Many sRNA-based technologies have been developed to protect plants from pathogens. **a** Artificial microRNA (amiRNA) approach. For example, the transgenic barley lines that carry a polycistronic amiRNA precursor construct (VirusBuster171) expressing three amiRNAs simultaneously under the control of a constitutive promoter, display enhanced resistance to *Wheat dwarf virus* (*WDV*) (Kis et al. 2016). **b** Short tandem target mimic (STTM) approach in modulating the activity of miRNAs. STTM is composed of two miRNA binding sites which have mismatches at the mRNA cleavage sites. The two mimic sequences are usually separated by a spacer linker. In soybean, inhibition of miR1507 as well as miR482 by STTM compromises their suppression of NBS-LRR genes (Bao et al. 2018). **c** Synthetic trans-acting siRNA approach. MiRNAs target TAS loci and produce phased, secondary siRNAs with the help of DCL4. Thus, *TAS* genes are engineered to express multiple synthetic ta-siRNAs (syn-tasiRNAs) that target multiple viruses at diverse genomic position. **d** Host-induced gene silencing (HIGS), spray-induced gene silencing (SIGS) and virus-induced gene silencing (VIGS). Based on that plant sRNAs can be transferred to organisms colonizing or feeding on the plant, scientists engineer transgenic plants which produce sRNAs targeting pathogen sequences to avoid infection. Meanwhile, VIGS, which use the virus expression vector as the medium, and SIGS, which directly use pathogen-gene-targeting dsRNAs or sRNAs, are two other strategies. These new approaches not only control plant disease but also have the advantages of simplicity, high specificity, flexibility and stability

Based on the above knowledge on the roles of small RNAs in plant immunity, many sRNA-based technologies have been developed to protect plants against pathogens, such as artificial microRNA (amiRNA) and synthetic *trans*-acting siRNA (syn-tasiRNA)-mediated RNAi, host-induced gene silencing (HIGS), spray-induced gene silencing (SIGS) and virus-induced gene silencing (VIGS) (Fig. 2a-d). These strategies are applied to breed crops with stable disease resistance or fine-tune the trade-off between plant immunity and yield.

amiRNA-induced silencing approaches are widely used to protect plants against viruses (Fig. 2a). *A. thaliana* transgenic plants which express modified miR159 precursor-based amiRNAs targeting two viral suppressors P69 and Hc-Pro simultaneously, exhibit resistance against both *Turnip yellow mosaic virus* (*TYMV*) and *Turnip mosaic virus* (*TuMV*) (Niu et al. 2006). Similarly, using Arabidopsis pre-miRNA159a as the backbone, overexpressing amiRNAs targeting the replicase gene of *Watermelon silver mottle virus* (*WSMoV*) in *N. benthamiana* enhance plant defense even 21 days post viral inoculation (Kung et al. 2012). These findings prove the possibility of employing the amiRNA approach to breed crops with broad-spectrum resistance to tospoviruses as well as other viruses (Mitter et al. 2016). The transgenic barley lines that carry a polycistronic amiRNA precursor construct (VirusBuster171) expressing three amiRNAs simultaneously under the control of a constitutive promoter, display enhanced resistance to *Wheat dwarf virus* (*WDV*) (Kis et al. 2016). Besides amiRNA technology, short tandem target mimic (STTM) technology is also used to repress the function of miRNAs (Fig. 2b). Inhibition of miR1507a, miR1507c, miR482a, miR168a and miR1515a by STTM compromise *Soybean mosaic virus* (*SMV*) infection efficiency in soybean (Bao et al. 2018). Tomato STTM482/2118b lines display enhanced resistance to infection with the oomycete and bacterial pathogens (Canto-Pastor et al. 2019). *TAS* genes are also engineered to express multiple synthetic ta-siRNAs (syn-tasiRNAs) that target multiple viruses at diverse genomic positions (Fig. 2c). For example, The Arabidopsis *TAS3a* gene is used as backbone to generate syn-tasiRNAs targeting the genome of *TuMV* and *Cucumber mosaic virus* (*CMV*). Transgenic Arabidopsis plants expressing these syn-tasiRNAs show elevated resistance to both viruses (Chen et al. 2016). In tomato, expression of a syn-tasiRNA construct that can produce four different syn-tasiRNAs against *Tomato spotted wilt virus* (TSWV) enhance plant antiviral resistance (Carbonell et al. 2019).

Besides RNAi technologies based on amiRNAs, STTM and syn-tasiRNAs, many new RNA-based approaches, such as HIGS (Nowara et al. 2010), VIGS (Cooper and Campbell 2017; Ranjan et al. 2018), and SIGS (Koch et al. 2016), have been developed to control plant disease by targeting host susceptibility factors, pathogen-derived RNAs or pathogen effectors (Fig. 2d). HIGS technology has been widely used to defend plants against fungal pathogens. Expressing sRNAs that target *Bc-DCL1* and *Bc-DCL2* in tomato silence *Bc-DCL* genes and attenuate fungal pathogenicity and growth, suggesting the bidirectional cross-kingdom RNAi and sRNA trafficking between plants and fungi (Wang et al. 2016b). Expressing CYP3 dsRNA in barley leaves produces sRNAs to silence fungal cytochrome P450, which inhibit the growth and alter fungal morphology of *F. graminearum* at the incubation site (Koch et al. 2013). In barley and wheat, the accumulation of dsRNAs or antisense RNA targeting fungal transcripts affects the development of the powdery mildew fungus *Blumeria graminis* (Nowara et al. 2010). Surprisingly, dsRNAs have efficacy to target pathogens even directly sprayed on plants, which is known as SIGS (Sang and Kim 2020). Foliar application of dsRNA is effective in reducing disease symptoms in *Brassica napus* infected by *Sclerotinia sclerotiorum* or *B. cinerea* (McLoughlin et al. 2018). Similar to HIGS, direct spraying detached barley leaves with *CYP3* dsRNAs prior to *F. graminearum* infection also effectively inhibits disease development (Koch et al. 2016). SIGS of *F. graminearum* AGO and DCL genes protects barley leaves from *F. graminearum* infection (Werner et al. 2020). Moreover, SIGS has been adopted to compromise *Verticillium wilt* in tomato (Song and Thomma 2018) and late blight disease in potato (Kalyandurg et al. 2021). It is needed to note that the efficiency of SIGS for disease control largely depends on the efficiency of RNA uptake by pathogens and the secondary amplification of siRNA machinery (Qiao et al. 2021; Song and Thomma 2018).

Another approach to enhance plant resistance to pathogens is VIGS which takes advantage of plant sRNAs-mediated antiviral defense mechanism. VIGS is nowadays widely used to engineer knockdown plants due to its easy to operate and high efficiency. VIGS mainly involves three steps: design vectors derived from viruses carrying viral target genes to be silenced, infect the plant hosts by *Agrobacterium tumefaciens* and silence the target genes to activate defense against pathogen infection (Becker and Lange 2010). The viral vectors used in VIGS are mainly derived from positive-strand RNA viruses such as *Potato virus X* (PVX), TMV, and *Tobacco rattle virus* (TRV) (Purkayastha and Dasgupta 2009). The *Barley stripe mosaic virus* (*BSMV*), a tripartite RNA virus, which can infect many important crops like barley, wheat, rice and maize, is also modified for VIGS (Purkayastha and Dasgupta 2009). Many studies have revealed that VIGS is a powerful tool for identifying functional genes that confer disease resistance in crops. For example, knocking-down *TaBON1* or *TaBON3* by VIGS enhances wheat disease resistance to powdery mildew by upregulating defense responses in wheat (Zou et al. 2018). The reduced expression of *TaDIR1-2* through the *BSMV*-mediated VIGS system contributes to the wheat defense response against *Puccinia striiformis* f. sp. *tritici* (Ahmed et al. 2017; Scofield et al. 2005). The role of agmatinecoumaroyl transferase *TaACT* in *Fusarium head blight* (*FHB*) resistance is validated by VIGS in wheat (Kage et al. 2017). Through VIGS strategy, the regulatory role of the TaMED25-TaEIL1-TaERF1 module in bread wheat defense against powdery mildew is identified (Liu et al. 2016). In tomato, reverse genetic studies using VIGS technology reveal that SlMAPKKKε, a positive regulator of cell death, is required for disease resistance against *Pst* and *Xanthomonas campestris* pv. *vesicatoria* (*Xcv*) (Melech-Bonfil and Sessa 2010).

## Concluding remarks and perspectives

Due to the ever-lasting arms race between plants and pathogens, there are no permanent disease control strategies which are absolute effective or completely without risk (including off-target effects, selection between ideal and specific target sites). As reviewed above, small RNAs mediate multilayer regulation in crop immune responses against pathogens, including post-transcriptional gene silencing by guiding mRNA cleavage/degradation or translational repression, transcriptional gene silencing by direct DNA methylation or chromatin modification (Fig. 1 and Table 1). A series of evidence indicate that these pathogen-responsive small RNAs may fine-tune or reprogram gene expression by silencing negative regulators or inducing positive regulators of immune responses.

**Table 1:** small RNAs involved in crop disease resistance

Pathogen		Crop	Small RNA	Influence (in plant resistance)	Target	Reference·
Fungi	*Magnaporthe oryzae*	rice	miR156	negative	SPL14	(Zhang et al. 2020)
	*Magnaporthe oryzae*	rice	miR160	positive	ARF16	
	*Magnaporthe oryzae*	rice	miR162	positive	DCL1a	(Li et al. 2020b)
	*Magnaporthe oryzae*	rice	miR164a	negative	OsNAC60	(Wang et al. 2018)
	*Magnaporthe oryzae*	rice	miR166	positive	EIN2	(Salvador-Guirao et al. 2018)
	*Magnaporthe oryzae*	rice	miR167d	negative	ARF12	(Zhao et al. 2020)
	*Magnaporthe oryzae*	rice	miR168	negative	AGO1	(Wang et al. 2021)
	*Magnaporthe oryzae*	rice	miR169	negative	NF-YAs	(Li et al. 2017)
	*Magnaporthe oryzae*	rice	miR319	negative	TCP21	(Zhang et al. 2018)
	*Magnaporthe oryzae*	rice	miR396	negative	OsGRFs	(Chandran et al. 2018)
	*Magnaporthe oryzae*	rice	miR398	positive	CSD1, CSD2, SODX	(Li et al. 2019a)
	*Magnaporthe oryzae*	rice	miR439	negative	/	(Lu et al. 2021)
	*Magnaporthe oryzae*	rice	miR812	positive	ACO3, CIPK10, LRR	(Campo et al. 2021)
	*Magnaporthe oryzae*	rice	miR7695	positive	OsNramp6	(Campo et al. 2013)
	*Magnaporthe oryzae*	rice	MITE1/2-siRNAs	positive	PigmS	(Deng et al. 2017)
	*Fusarium fujikuroi*	rice	miR166	positive	EIN2	(Salvador-Guirao et al. 2018)
	*Rhizoctonia solani*	rice	siR109944	negative	FBL55	(Qiao et al. 2020)
	*Blumeria graminis* f. sp.*hordei*	Barley	miR398	negative	HvSOD1	(Xu et al. 2014)
	*Blumeria graminis* f. sp*.hordei*	Barley	miR9863	negative	MLA1	(Liu et al. 2014)
	*Verticillium dahlia*	cotton	miR159	positive	HiC-15	(Zhang et al. 2016c)
	*Verticillium dahlia*	cotton	miR166	positive	Clp-1	(Zhang et al. 2016c)
	*Verticillium dahlia*	cotton	miR477	positive	CBP60A	(Hu et al. 2020)
	*Puccinia striiformis* f. sp. *tritici*	wheat	miR408	negative	CLP1	(Feng et al. 2013)
	*Fusarium graminearum*	wheat	miR1023	positive	FGSG_03101	(Jiao and Peng 2018)
Oomycete	*Phytophthora infestans*	potato	miR160	unknown	ARF10	(Natarajan et al. 2018)
	*Phytophthora sojae*	soybean	miR393	positive	/	(Wong et al. 2014)
	*Phytophthora sojae*	soybean	miR1507	unknown	Glyma04g29220, Glyma06g39720, Glyma06g39720	(Wong et al. 2014)
	*Phytophthora sojae*	soybean	miR219	unknown	Glyma06g39720, Glyma01g06750	(Wong et al. 2014)
	*Phytophthora sojae*	soybean	miR1510	negative	Glyma.16G135500	(Cui et al. 2017)
Bacterial	*Xanthomonas oryzae* pv. *Oryzae*	rice	miR156	negative	IPA1, OsSPL7	(Liu et al. 2019)
	*Xanthomonas oryzae* pv. *Oryzae*	rice	miR159	unknown	GAMYB1, OsLRR-RLK2	(Zhao et al. 2015b)
	*Xanthomonas oryzae* pv. *Oryzae*	rice	TE-siR815	negative	ST1	(Zhang et al. 2016a)
	*Xanthomonas oryzae* pv. *Oryzae*	rice	miR1876	negative	NBS8R	(Jiang et al. 2020)
	*Bacillus velezensis FZB42*	maize	miR169	unknown	NF-Y	(Xie et al. 2019)
			miR395	unknown		
Virus	*Tobacco rattle virus (TRV)/Cucumber mosaic virus (CMV)*	tomato	miR482	negative	LRR1, LRR2	(Shivaprasad et al. 2012)
	*Rice black streaked dwarf virus (RBSDV)*	rice	miR393	negative	TIR1	(Zhang et al. 2019)
	*Rice stripe virus (RSV)*	rice	miR168	negative	AGO1	(Du et al. 2011)
	*Rice stripe virus (RSV)*	rice	miR444	positive	RDR1, MADS	(Wang et al. 2016a)
	*Rice stripe virus (RSV)*	rice	miR528	negative	AO	(Wu et al. 2017) (Yao et al. 2019)
	*Tobacco mosaic virus (TMV)*	tobacoo	miR6019, miR6020	negative	N	(Li et al. 2012)
	*Turnip mosaic virus (TuMV)*	*Brassica napus*	miR1885	negative	BraTIR1, BraTNL1	(Cui et al. 2020)
	*Soybean mosaic virus (SMV)*	soybean	miR168	negative	AGO1	(Bao et al. 2018)
	*Soybean mosaic virus (SMV)*	soybean	miR1515	/	DCL2	
	*Soybean mosaic virus (SMV)*	soybean	mIR1507, miR482	negative	NBS-LRR	

Despite the advantages of simplicity, high specificity, flexibility and stability, there are some limitations of RNAi-based approaches. For example, a major hurdle in the practical application of SIGS is the rapid degradation of naked RNAs. To overcome this problem, nanomaterials such as chitosan-complexed single-walled carbon nanotubes (Demirer et al. 2019; Kwak et al. 2019) and layered double hydroxide (LDH) clay nanosheets (Qiao et al. 2021), may be used to deliver and stabilize sRNAs. These new approaches not only facilitate biomolecule transport into plant cells with high efficiency and without toxicity or tissue damage but also protect RNA cargo from nuclease degradation. Other candidate protective delivery systems such as artificial extracellular vesicle (EVs) or liposomes mimic plant EV may also be used (Regente et al. 2017). However, our knowledge of plant EV is rather limited. Whether artificial EVs can function in vivo still needs to be investigated. Despite efficient dsRNA uptake in many fungal plant pathogens have been achieved, the efficiency of dsRNAs uptake in other fungi still need to be resolved (Qiao et al. 2021; Rosa et al. 2018). Moreover, strategies for control bacteria-induced diseases are rather limited.

To breed non-genetically modified (GMO), disease-tolerant crops, SIGS will be a promising technology to enhance crop resistance to disease. sRNAs-based strategies may be synergistically applied with other molecular approaches. Combined with the use of newly developed multi-transgene stacking toolkit containing marker/marker-excision cassette (Zhu et al. 2017), HIGS may be optimized to silence multiple pathogens through various RNA constructs targeting different genes in pathogens. Moreover, multiple viral vectors-based transient reprogramming has been used to trigger alterations of agronomic traits, such as flowering time, plant height or drought tolerance (Torti et al. 2021). Similar viral vectors-based RNAi may be developed to manage plant pathogen pandemics. To date, there are almost no RNAi-based field applications for managing plant diseases caused by bacteria and fungi, although there are already many field trials and even approval for RNAi-based targeting of plant pathogens (Rosa et al. 2018). More economic, high efficiency, low toxicity agricultural development will be the perpetual goals. RNAi-based technology will play a profound role in protecting plant from pathogens in the future.

## Data Availability

Not applicable.

## Abbreviations

Small RNAs (sRNAs): A class of 18-30 nt, non-coding RNAs, including microRNAs and small interfering RNAs in plants, which modulate gene expression and maintain the genome stability
Pathogen-associated molecular patterns (PAMPs): Small molecules from microbes that can be recognized by host pattern recognition receptors
Pattern recognition receptors (PRRs): A class of receptors responsible for recognizing the PAMPs from microbes
Pathogen-associated molecular pattern (PAMP)-triggered immunity (PTI): The immune responses that are initiated by the recognition of PAMPs by PRRs
Effector-triggered immunity (ETI): A cascade of defense responses that are initiated by the direct or indirect recognition of pathogen effectors by host resistance (R) proteins
RNA-induced silencing complex (RISC): A complex containing ARGONAUTE (AGO) proteins, single-stranded small RNAs and other proteins, which can silence target gene expression by mRNAs cleavage, translational inhibition, or DNA methylation-mediated transcriptional inhibition
RNA-directed DNA methylation (RdDM): The de novo cytosine methylation in plants that is guided by small interfering RNAs (siRNAs)
Systemic acquired resistance (SAR): The systemic immune responses triggered by an initial localized infection, which confer long-lasting and broad-spectrum protection in plants
Artificial microRNAs (amiRNAs): A class of artificial small RNAs generated from synthetic miRNA precursors using endogenous pre-miRNA as the backbone
Synthetic *trans*-acting siRNAs (syn-tasiRNAs): Artificial siRNAs produced from the engineered *TAS* locus
Short tandem target mimic (STTM): A potent technology for blocking the activity of small RNAs, which triggers the degradation or sequestration of target small RNAs.
Host-induced gene silencing (HIGS): An RNAi-based technology in which sRNAs are produced from dsRNAs expressed in hosts to induce silencing of pathogen genes, which protect plants from pathogens
Spray-induced gene silencing (SIGS): A newly developed technology used to increase plant resistance against pathogens by direct spraying dsRNAs on plants
Virus-induced gene silencing (VIGS): An efficient method that is applied to block host gene expression through modified virus-based vectors carrying sequences derived from corresponding host genes
